# Automated Spectrophotometric Assays for the Measurement of Ammonia and Bicarbonate in Saliva of Horses: Analytical Validation and Changes in Equine Gastric Ulcer Syndrome (EGUS)

**DOI:** 10.3390/metabo14030147

**Published:** 2024-02-28

**Authors:** Alberto Muñoz-Prieto, Eva Llamas-Amor, María Dolores Contreras-Aguilar, Ignacio Ayala, María Martín Cuervo, José Joaquín Cerón, Sanni Hansen

**Affiliations:** 1 Interdisciplinary Laboratory of Clinical Analysis (Interlab-UMU), Regional Campus of International Excellence ‘Campus Mare Nostrum’, University of Murcia, Campus de Espinardo s/n, 30100 Murcia, Spain; eva.llamasa@um.es (E.L.-A.); mariadolores.contreras@um.es (M.D.C.-A.); iayape@um.es (I.A.); jjceron@um.es (J.J.C.); 2 Department of Animal Medicine, Faculty of Veterinary, University of Extremadura, Avda de la Universidad s/n, 10003 Cáceres, Spain; mariamc@unex.es; 3 Section Medicine and Surgery, Department of Veterinary Clinical Sciences, University of Copenhagen, Agrovej 8, 2630 Taastrup, Denmark; sannih@sund.ku.dk

**Keywords:** ammonia, bicarbonate, EGUS, saliva, horses

## Abstract

Ammonia (NH_3_) and bicarbonate (HCO_3_) have been related to gastric ulcers in humans. Ammonia is considered a possible cause of gastric ulcers, whereas bicarbonate has a protective function. The presence of ulcers in the stomach of horses is defined as Equine Gastric Ulcer Syndrome (EGUS), which is a frequent disease in this species, and it has been associated with changes in saliva composition, such as in analytes related to inflammation, immune system and oxidative stress. The objectives of this study were (1) to perform an analytical validation of two automated spectrophotometric assays, one for ammonia and one for bicarbonate, in the horses’ saliva and (2) to evaluate their possible variations with EGUS. Analytical validation of the automated assays for ammonia and bicarbonate in the saliva of horses showed that both assays were precise and accurate. In addition, significantly higher values of ammonia and lower values of bicarbonate were found in the saliva of horses with EGUS compared to healthy horses. It can be concluded that ammonia and bicarbonate can be measured in the saliva of horses and that ammonia increases and bicarbonate decreases in this sample type could be related to the presence of EGUS in this species.

## 1. Introduction

Ammonia (NH_3_) is a ubiquitous metabolite that can be generated from different precursor molecules, such as arginine via arginine deaminase or urea through urease [1]. In people, the measurement of ammonia has been studied in saliva due to the advantages of this fluid since it is easier to collect and is less invasive than whole blood and can provide information about diseases in which there is an increase in concentrations of this ion [2]. It has been shown to be a key biomarker for a wide variety of diseases, such as hepatic and chronic kidney failure and cancers. It also has relevance to the oral health research area because it has been related to caries development [2]. The origin of ammonia in saliva is unclear, but one mechanism could be the diffusion from blood as ammonia passes easily across membranes [3]. For example, in sheep, similar changes in ammonia in saliva and blood has been observed [4]. Ammonia can be measured in the saliva of humans by different methods, such as spectrophotometric assays, ion-selective electrodes [5] and nuclear magnetic resonance spectroscopy [2].

Bicarbonate (HCO_3_) is a widely diffused compound in the organism with a major buffering function. In saliva, bicarbonate is partly derived from CO_2_, which is converted to bicarbonate in the presence of carbonic anhydrase (CA) in the salivary glands [6,7]. The concentration of bicarbonate in saliva could further be influenced by blood values and certain stimuli, such as pilocarpine administration, which has been found to increase its production in the salivary gland [6,7]. In horse saliva, bicarbonate has been measured with ion-specific electrodes, and it has been related to the neutralization of the acid produced by the oral microorganisms [8]. 

Ammonia is considered a possible cause of gastric ulcers, and it is documented that gastric juice ammonia correlates with the severity of gastritis in people [9]. Ammonia impairs mitochondrial and cellular respiration and energy metabolism in the human stomach cells, and it decreases mucosal cell viability, subsequently leading to mucosal damage [10]. In addition, in rats, ammonia can suppress cell cycling of the regenerative epithelium and fibroblasts and produce direct toxicity in gastric cells. Furthermore, the presence of this ion leads to a delay in the healing of peptic ulcers [11]. 

Bicarbonate has been described to have a protective function against gastric ulcers. The gastroduodenal mucosa’s surface epithelial cells are shielded from acid-induced harm through a protective layer of mucus and bicarbonate. The mucus acts as an effective hindrance to the diffusion of hydrogen ions [12]. Bicarbonate is produced by the gastric and duodenal mucosal cells and regulates the pH of the mucus, ensuring an optimal range for enhancing its barrier properties and acting as an important mucosal defense mechanism [13]. Bicarbonate has been used to treat peptic ulcers together with omeprazole [14]. In addition, stronger salivary bicarbonate secretion could be a major factor protecting the upper esophageal mucosa against acid exposition [15].

The presence of ulcers in the stomach of horses is defined as Equine Gastric Ulcer Syndrome (EGUS), which is a frequent disease in this species [16]. Two entities have been observed in this syndrome: the equine glandular gastric disease (EGGD) and the equine squamous gastric disease (ESGD) [17]. Previous work has associated EGUS with changes in saliva composition, especially in analytes related to inflammation, the immune system and oxidative stress [18]. However, to the author’s knowledge, no reports exist that evaluate ammonia and bicarbonate in horse saliva related to EGUS. The hypotheses of this study were (1) that ammonia and bicarbonate could be measured in the saliva of horses by automated spectrophotometric assays commercially available and (2) that these two analytes could change in saliva concentration in horses with EGUS. The objective of this study was to perform an analytical validation of two automated spectrophotometric assays, one for ammonia and one for bicarbonate, in the saliva of horses and further to evaluate the possible variations in these analytes with EGUS.

## 2. Materials and Methods

### 2.1. Animals

The horses analyzed in the research were sourced from the Large Animal Teaching Hospital at the University of Copenhagen and the Veterinary Teaching Hospital of the University of Extremadura, covering the period from August 2020 to August 2023. 

For all horses, a comprehensive evaluation was conducted, encompassing medical history, clinical assessments (including weight, body condition score (BCS) evaluated on a nine-point scale [19], heart rate, respiratory rate, rectal temperature, color of mucous membranes, capillary refill time, and borborygmus), as well as hematological and biochemical analyses. Gastroscopy was conducted on all subjects following a 12 h fasting period, as previously outlined [20]. Images acquired through gastroscopy were employed to identify EGSD and EGGD, adhering to the ECEIM Consensus Statement [3,21]. To diagnose ESGD, an animal was deemed positive if it scored ≥1 on the 0–4-point ESGD grading scale. To diagnose EGGD, an animal was deemed positive if a score of ≥1 on a 0–4-point EGGD grading scale was reached [22]. Depending on the case, supplementary diagnostic procedures were undertaken, such as rectal examination, transabdominal ultrasonography, abdominocentesis or exploratory laparotomy.

Based on the outcomes of clinical assessments and diagnostic examinations, horses were categorized into three distinct groups:

1. EGUS group. This group included horses displaying clinical manifestations (such as riding difficulties, weight loss, and alterations in temperament and/or pain behaviors) and gastroscopy findings consistent with EGUS, as per the previously specified criteria [3]. This group was further subdivided into ESGD (assessed using the 4-point scale mentioned above), EGGD (assessed using the 4-point scale mentioned above) or both ESGD and EGGD. Only animals diagnosed solely with EGUS and without evidence of other alterations were included in this group.
2. Non-EGUS group. This group included animals with gastrointestinal signs that could raise suspicion for EGUS but with no compatible images at gastroscopy. Supplementary diagnostic procedures were conducted, as indicated above, to identify the particular disease.
3. Healthy horses. This group comprised horses admitted for castration or routine health examinations. The animals exhibited no clinical indications of abdominal pain or any other abnormalities during the physical examination. Hematological and biochemical findings remained within the reference values, and there were no signs of EGUS either before or after the gastroscopy examination.

### 2.2. Sampling

As mentioned earlier, saliva samples were gathered from all horses prior to intravenous sedation, and gastroscopy was conducted immediately after the horses were positioned in the examination stand [18,23]. Saliva was collected using a sponge, which was then placed into a Salivette tube. The tubes were kept at 4 °C until reaching the laboratory within 20 min of collection. Upon arrival, centrifugation at 3000× *g* for 10 min was performed to extract saliva, which was subsequently preserved at −80 °C until analysis. All saliva samples were frozen within 1 h after collection, which is defined as the period in which ammonia analysis should be made to avoid artefactual increases [5,24].

### 2.3. Ammonia and Bicarbonate Assays

Ammonia was determined through an enzymatic colorimetric assay (Spinreact, Girona, Spain). The principle of the assay is that ammonia combines with α-ketoglutarate and NADPH in the presence of glutamate dehydrogenase (GLDH) to yield glutamate and NADP+. The corresponding decrease in absorbance at 340 nm is proportional to the plasma ammonia concentration. Bicarbonate was measured with an enzymatic-colorimetric assay (Biosystem S.A., Barcelona, Spain) based on the consumption of NADH analogous-cofactor by the carbon dioxide (CO_2_) present in the sample. Both assays were adapted to an automated biochemical analyzer Olympus AU400 (Beckman Coulter, Brea, CA, USA).

The saliva samples of horses used in this study were analyzed in the same batch and stayed less than one hour in an automated analyzer in order to avoid possible artefactual changes [5]. Furthermore, at the end of the analysis of all samples, the first five sample analyses of the batch were measured and gave values less than 10% of the variation.

### 2.4. Validation Study of Ammonia and Bicarbonate Assays

The ammonia and bicarbonate assays were validated in horse saliva samples using aliquots of horses’ saliva with different EGUS grades. The validation of the assays was assessed as follows:

- Precision: directly evaluated by the intra- and inter-assay coefficients of variation (CVs) using saliva samples with high and low concentrations of ammonia and bicarbonate.
- Accuracy: assessed through linearity after dilution studies with ultrapure water of saliva samples with high concentrations of ammonia and bicarbonate, respectively.
- Lower limit of quantification (LLQ): calculated as the lowest concentration of ammonia and bicarbonate that the assays could determine with an intra-assay CV < 20%.
- Limit of detection (LD): defined as the lowest concentration of ammonia and bicarbonate that the assays were capable of discerning a specimen with zero value (ultrapure water), determined by calculating the mean value plus 3 standard deviations from 12 replicate measurements of ultrapure water.

### 2.5. Stability Study of Ammonia and Bicarbonate in Horse Saliva

The impact of varying storage temperature conditions over time was assessed for the determination of ammonia and bicarbonate levels in equine saliva. Five saliva samples were used from five horses at the Teaching Farm of the University of Murcia, and the following time points were measured: T0: immediately after arrival to the laboratory in no more than 20 min after collection. In addition, at the laboratory, each sample was aliquoted in four subsamples: one was kept at room temperature, one was refrigerated at 4 °C, one frozen at −20 °C, and one frozen at −80 °C. Ammonia and bicarbonate were determined before storage (T0) and after 6 h, 24 h, 48 h and 30 days. 

### 2.6. Ammonia and Bicarbonate Concentrations in the Saliva of Horses with Equine Gastric Ulcer Syndrome

The changes in the ammonia and bicarbonate concentrations were compared in the different groups of horses included in the study as indicated in Section 2.1.

### 2.7. Statistical Analysis

Data were assessed for normality using the Kolmogorov–Smirnov test, giving a non-parametric distribution. Therefore, a non-parametric test was selected to perform a comparative analysis. First, the Mann–Whitney test was employed to assess differences between all horses with EGUS and controls and between horses with EGUS and non-EGUS. Then, the Kruskal–Wallis test was used to compare the means between the different EGUS groups, including controls. Results were expressed as median and interquartile range (IQR). A *p* < 0.05 was considered significant.

To assess stability, the imprecision of the assay was evaluated using the intra-assay coefficient of variation (Intra-CV). After each measurement point, the loss and recovery of ammonia and bicarbonate were calculated as percentages from the initial analysis (T0 as 100% of each analyte concentration of saliva sample). Measurements were calculated using the following formula: (T − T0) × 100/T0. Therefore, ammonia and bicarbonate were deemed stable if alterations observed in the stored samples did not surpass the acceptable significant change limit (SCL) for the assay. The SCL was defined as SCL = 100% ± 2 × Intra-CV [25]. A two-way ANOVA test of repeated measures, along with Dunnett’s multiple comparison test, was employed to determine the statistical significance of the percentage changes observed in analyte levels over time at various temperatures. Changes beyond the Significant Change Limit (SCL) and exhibiting significant differences from T0 were regarded as indicative of unacceptable stability under the specified storage conditions.

The computations were conducted using GraphPad Prism 8 (GraphPad Software, San Diego, CA, USA) and SPSS (IBM SPSS Statistics for Windows, Version 28.0.1. IBM Corp, Endicott, NY, USA) statistical packages, with the significance level established at *p* < 0.05.

## 3. Results

### 3.1. Description of Horses Included

A total of 122 horses were included in the study. The healthy group consisted of 31 animals (20 mares, 11 geldings) representing various breeds, with a median age of 13.6 years (range: 3–23) and a Body Condition Score (BCS) of 5.4 (range: 4–8). The EGUS group comprised a total of 71 horses (36 mares, 35 geldings) from diverse breeds, with a median age of 11.6 years (range: 4–24) and a BCS of 5.2 (range: 4–8). This group included 21 horses with ESGD, 25 with EGGD, and 25 with both ESGD + EGGD. A group of horses with other intestinal diseases different from EGUS was composed of 20 animals of diverse breeds (11 mares, 9 geldings) with a median of 11.5 (range 2–20) years old and a BCS of 5.2 (range 4–8). Information about the diseases of these animals is indicated in Supplementary Table S1. There were no statistically significant differences when comparing age and BCS in the groups.

### 3.2. Analytical Validation of the Ammonia and Bicarbonate Assay in Horse Saliva

The ammonia assay showed mean intra- and inter-assay CVs of 5.68 and 9.94%, while the bicarbonate assay displayed mean intra- and inter-assay CVs of 7.68% and 5.69%. 

**Figure 1:**
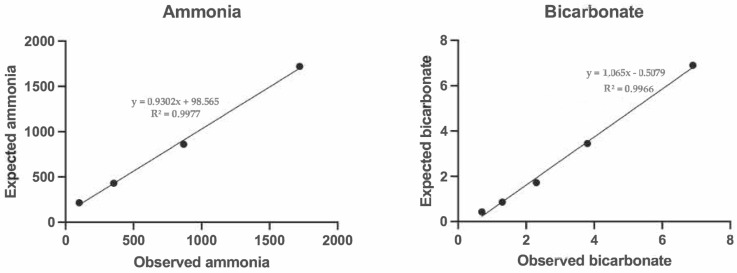
Linearity under dilution of the ammonia and the bicarbonate assay in horse saliva.

The serial dilution of saliva samples displayed linear regression equations, exhibiting a coefficient of correlation approaching 1 for ammonia and bicarbonate (Figure 1). The LLQ was set at 0.01 mmol/L for salivary CALP, and the LD of the assay could not be calculated since all measurements with ultrapure water gave negative values.

### 3.3. Stability of Ammonia and Bicarbonate in Horse Saliva

**Figure 2:**
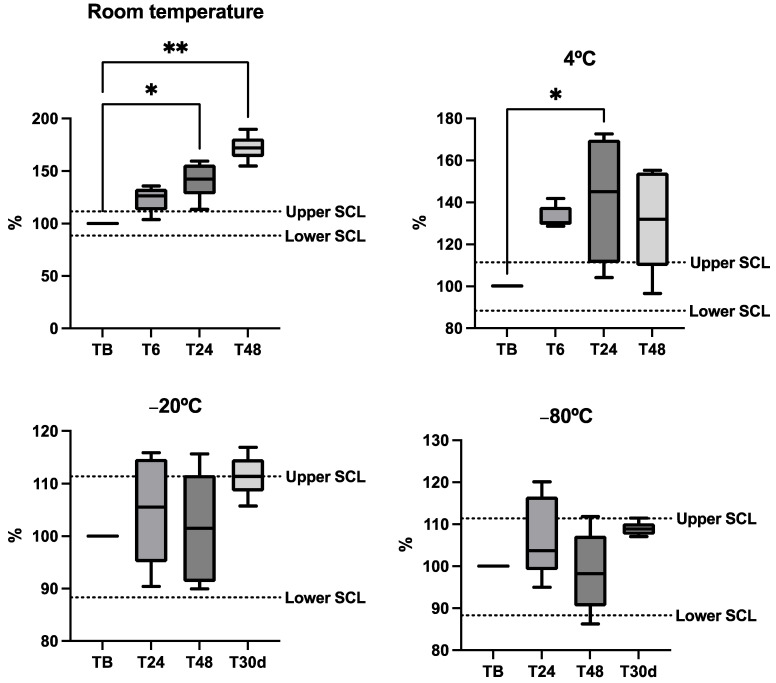
Stability plots of the ammonia in five horse saliva samples kept at different temperatures. Results are expressed in percentages of the baseline concentration (fresh sample = 100% concentration). Each aliquot was stored at different temperatures and measured in fresh conditions (TB) after 6 h, 24 h, 48 h and 30 days (T30d). The dotted lines indicate the significant change limit (SCL) acceptable for the assay, which was defined as SCL = 100% ± 2 × Intra-CV. The plot shows the median (line within the box), 10th and 90th percentiles (box), and the minimum and maximum values (whisker). Asterisks indicate significant differences from TB (*: *p* < 0.05; **: *p* < 0.01).

Ammonia stability in horse saliva is shown in Figure 2. No statistically significant differences between the different storage conditions were observed in any case during the first 6 h post-obtention, regardless of the temperature at which they were stored. However, median concentrations from samples stored at room temperature and 4 °C were outside of the SCL, being higher than the basal values after 6 h, and also showed significant changes compared with basal values after 24 h both at room temperature and 4 °C and after 48 h only at room temperature. When samples were frozen at −80 °C, they showed stable median concentrations during the complete stability study of 30 days with values within the SCL.

**Figure 3:**
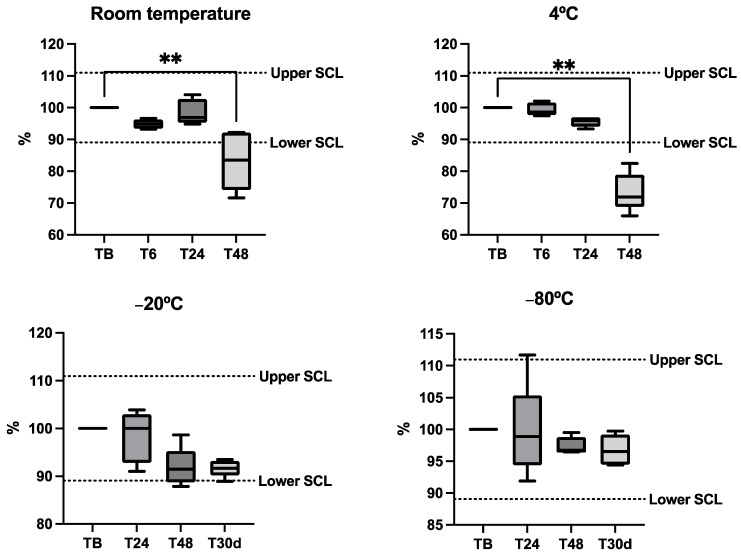
Stability plots of the bicarbonate in five horse saliva samples kept at different temperatures. Results are expressed in percentages of the baseline concentration (fresh sample = 100% concentration). Each aliquot was stored at different temperatures and measured in fresh conditions (TB) after 6, 24, 48 h and 30 days (T30d). The dotted lines indicate the significant change limit (SCL) acceptable for the assay, which was defined as SCL = 100% ± 2 × Intra-CV. The plot shows the median (line within the box), 10th and 90th percentiles (box), and the minimum and maximum values (whisker). Asterisks indicate significant differences from TB (**: *p* < 0.01).

Bicarbonate stability in horse saliva is shown in Figure 3. Bicarbonate concentrations in aliquot samples were within the SCL during 24 h after obtention with the independence of the storage temperature and did not show significant variations during this time. After 48 h, bicarbonate levels decreased, falling under the SCL at room temperature and at 4 °C, showing significant reductions in these conditions. 

### 3.4. Changes in Ammonia and Bicarbonate in Equine Gastric Ulcer Disease

**Figure 4:**
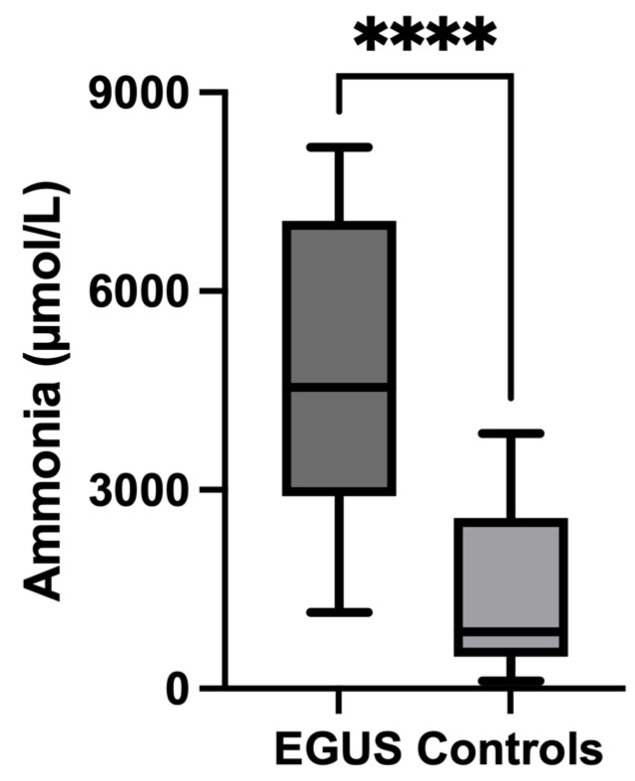
Ammonia concentrations in the saliva of horses with Equine Gastric Ulcer Syndrome (EGUS) and healthy horses (Control). The median values are represented by the lines, the 10–90 percentiles by the boxes, and the range by the whiskers. **** *p* < 0.0001.

**Figure 5:**
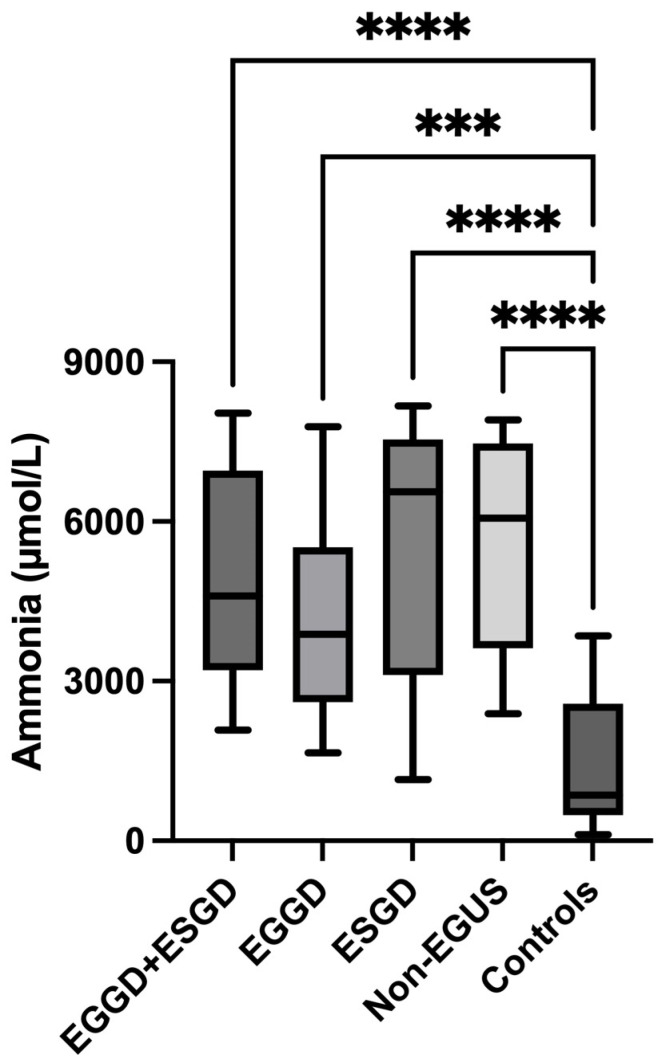
Comparison of ammonia concentrations in the saliva of horses with Equine Glandular Gastric Disease (EGGD) and Equine Glandular Gastric Disease (ESGD) at the same moment (EGGD + ESGD), horses with only EGGD, horses with only ESGD, horses with gastrointestinal signs but negative to Equine Gastric Ulcer Syndrome (Non-EGUS), and healthy horses (Controls). The median values are represented by the lines, the 10–90 percentiles by the boxes and the range by the whiskers. *** *p* < 0.001, **** *p* < 0.0001.

**Figure 6:**
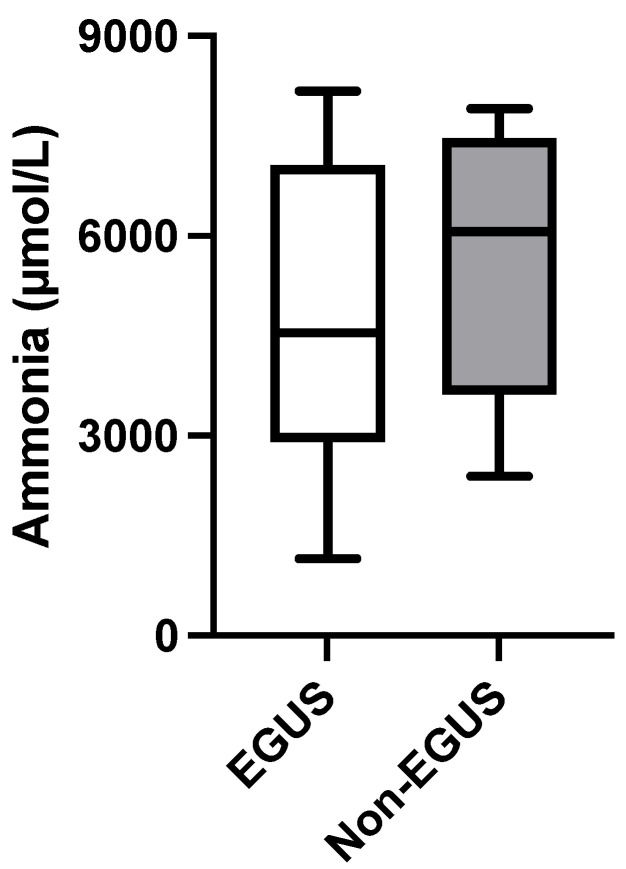
Ammonia concentrations in the saliva of horses with Equine Gastric Ulcer Syndrome (EGUS) and horses with gastrointestinal signs but negative to EGUS (Non-EGUS). The median values are represented by the lines, the 10–90 percentiles by the boxes and the range by the whiskers.

Ammonia levels were significantly higher in the saliva of horses with EGUS (4546 µmol/L, IQR = 2906–7060) compared to healthy horses (855.6 µmol/L, IQR = 485.5–2575) (*p* < 0.0001) (Figure 4). When separating per type of EGUS, concentrations of ammonia in horses with EGGD (3884 µmol/L, IQR = 2605–5517) (*p* < 0.001) and with ESGD 6555 µmol/L, IQR = 3115–7538) (*p* < 0.0001) were significantly higher than healthy horses. Furthermore, those horses with gastrointestinal signs but negative to EGUS showed higher values of ammonia (6061 µmol/L, IQR = 3620–7466) compared with healthy horses (*p* < 0.01) (Figure 5). No statistical differences were found in ammonia concentrations in horses with gastrointestinal signs but without EGUS (6061, IQR = 3620–7466) compared to horses with EGUS (4546 µmol/L, IQR = 2906–7060) (*p* = 0.202) (Figure 6).

**Figure 7:**
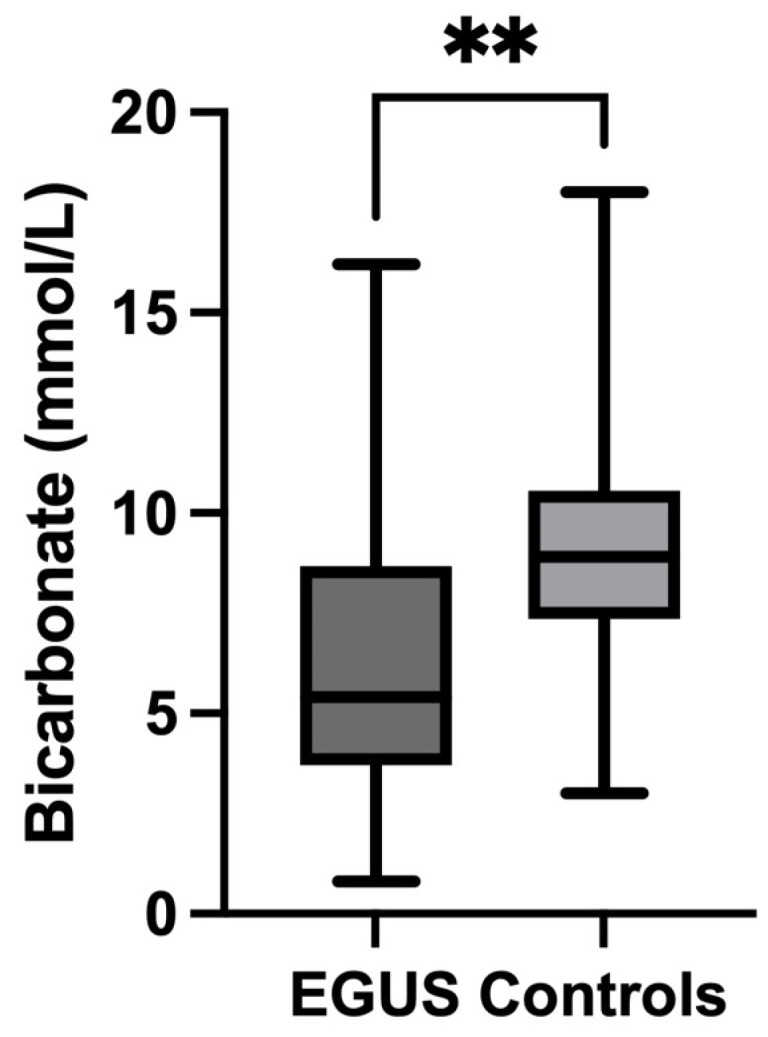
Bicarbonate concentrations in the saliva of horses with Equine Gastric Ulcer Syndrome (EGUS) and healthy horses (Control). The median values are represented by the lines, the 10–90 percentiles by the boxes and the range by the whiskers. ** *p* < 0.001.

**Figure 8:**
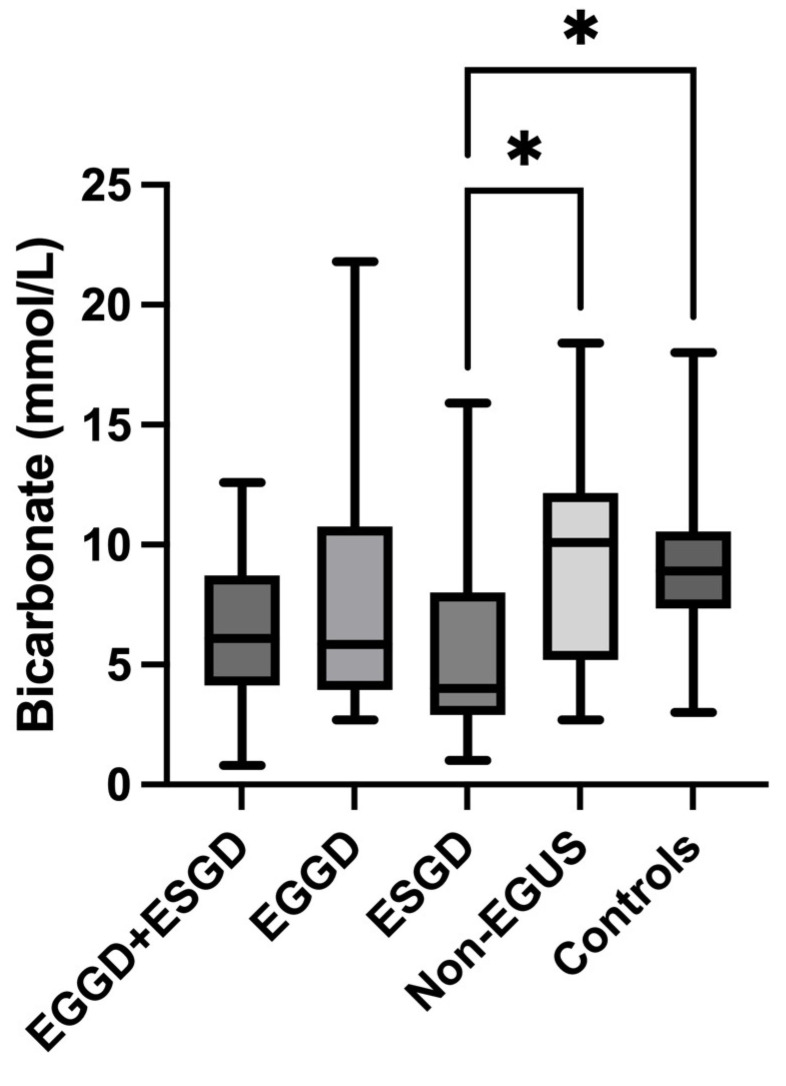
Comparison of bicarbonate concentrations in the saliva of horses with Equine Glandular Gastric Disease (EGGD) and Equine Glandular Gastric Disease (ESGD) (EGGD + ESGD), horses with only EGGD, horses with only ESGD, horses with gastrointestinal signs but negative to Equine Gastric Ulcer Syndrome (Non-EGUS) and healthy horses (Controls). The median values are represented by the lines, the 10–90 percentiles by the boxes and the range by the whiskers. * *p* < 0.01.

**Figure 9:**
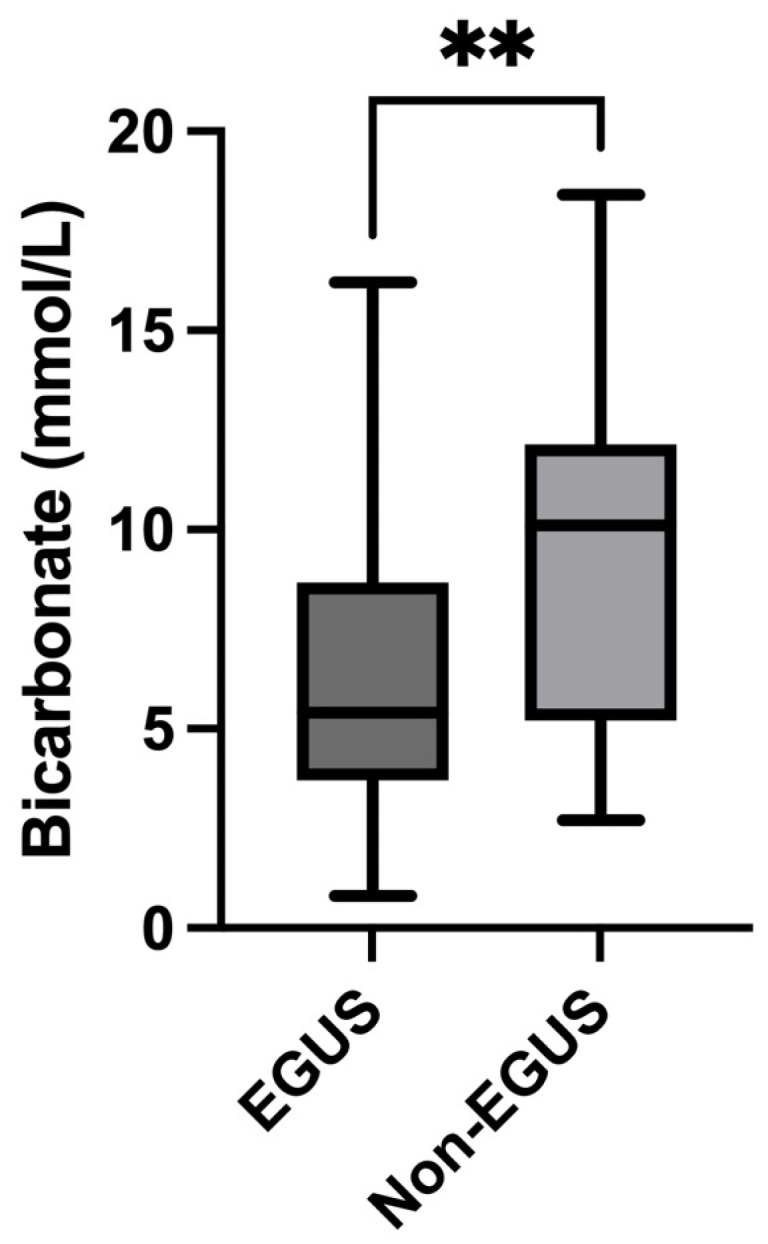
Bicarbonate concentrations in the saliva of horses with Equine Gastric Ulcer Syndrome (EGUS) and horses with gastrointestinal signs but negative to EGUS (Non-EGUS). The median values are represented by the lines, the 10–90 percentiles by the boxes and the range by the whiskers. ** *p* < 0.001.

In the case of bicarbonate, all horses with EGUS showed significantly lower salivary concentrations (5.4 mmol/L, IQR = 0.8–8.67) compared to controls (8.9 mmol/L, IQR = 7.35–10.55) (*p* = 0.002) (Figure 7). The comparison of the different types of EGUS indicated a decreased concentration of bicarbonate in all types of EGUS, but only in ESGD (4 mmol/L, IQR = 2.9–8) were significant changes compared to controls observed (8.9 mmol/L, IQR = 3–10.55) (Figure 8). Additionally, horses with gastrointestinal signs but without EGUS showed significantly higher bicarbonate concentrations (10.1 mmol/L, IQR = 5.2–12.15) than horses with EGUS (5.4, IQR = 3.7–8.67) (*p* = 0.009) (Figure 9).

## 4. Discussion

In this report, two automated assays for the measurement of ammonia and bicarbonate in horse saliva were analytically validated and increases in ammonia and decreases in bicarbonate were described in the saliva of horses with EGUS compared to healthy controls.

The methods used in this study were precise and accurate according to the criteria used for the analytical validation of assays [26] and, therefore, can be used for the measurement of ammonia and bicarbonate in the saliva of horses. These assays are commercially available, and although in this report were performed in an automated analyzer, they can also be adapted to ELISA plates or manual spectrophotometric assays. A similar enzymatic assay for ammonia, but not automatized, has been validated in human saliva with low imprecision and high accuracy [5]. These authors also validated other assays used for the measurement of ammonia in saliva, such as the Indophenol method or by electrodes [5]. Bicarbonate has been previously analyzed using an ion-specific electrode in the saliva of horses [8]. The method reported in our study can be an alternative for those laboratories in which electrodes are not available.

The stability trial performed in our report showed that saliva samples should be stored at −80 °C in order to prevent artifactual increases in ammonia and decreases in bicarbonate. This is in line with previous reports made in humans [5] in which increases in ammonia were found after a few hours in samples stored at room temperature or refrigerated. Similar results have been obtained in other sample types, such as cerebrospinal fluid, where there was no change in ammonia when stored at −20 °C for at least 1 month, but when stored at 4 °C, analysis should be performed within 2 days. In addition, storage at room temperature results in artificially rapid increased ammonia and should be avoided [27]. Based on these data, if samples cannot be measured immediately after collection, they should be stored preferably at −80 °C.

In saliva, an increased amount of ammonia was found in EGUS horses, as well as ESGD and EGGD, compared to healthy controls. Ammonia can produce direct damage in rat stomach with an alteration in the integrity and microscopic damage in the gastric mucosa. [28]. Ammonia inhibits oxygen consumption of cells and mitochondria of the gastric mucosa, leading to an impairment of energy metabolism and decreased mucosal cell viability, as well as the development of mucosal damage [10].

Further studies should be undertaken to clarify the reason for the increase in ammonia in EGUS. Ammonia can be produced from urea catalyzed by the enzyme urease, an enzyme presents in some bacteria species, such as *Helicobacter pylori*, described in human gastric ulcers [28], although in the case of horses, it seems that these bacteria are not involved in EGUS. However, ammonia can also be produced without the need for bacteria and urease by increased activity of the enzyme glutaminase, known to increase with inflammation [29]. Glutamine deamination, produced by the activity of glutaminase, has a central role in the immune pathogenesis of celiac disease and has been found to be implicated in autoimmune responses caused by various toxic substances and different pathogens [30]. Inflammation and autoimmune response could be involved in the EGUS pathogenesis but also in several other diseases with similar clinical signs as EGUS. An increase in glutaminase could be the reason for the increases in ammonia in EGUS, as well as the group included with clinical signs compatible with EGUS but without an EGUS diagnosis found in this study. 

The origin of the increased ammonia in saliva in this study is unclear, but one mechanism could be the diffusion from blood or other tissues or organs since ammonia passes easily across membranes [3]. It could be postulated that the increased ammonia in saliva could be influenced by the increase in gastric ammonia associated with gastric ulcers. In addition, it could be a local consequence of the gastric ulcer, as a local production of ammonia by the microflora in the oral cavity of humans has been found [31].

In humans, gastric bicarbonate secretion was reduced in patients with gastric ulcers, and it was indicated that the impairment of the mucus-bicarbonate barrier on the surface of the gastric mucosa is an important pathogenetic factor in the development of gastric ulcer [32,33]. The reduction in bicarbonate in saliva could possibly play a role in the development of gastric ulcers since it was indicated that the bicarbonate of the saliva could directly decrease gastric acidity and protect stomach mucosae, thereby reducing the risk of gastric ulceration [34]. 

In line with human reports, in our study, a decrease in saliva bicarbonate was found in horses with EGUS compared to healthy controls. An even more interesting, the group of horses with ESGD, a disease of the squamous part of the stomach, had the lowest amount of bicarbonate. The pathophysiology of squamous ulceration is due to a breakdown of the barrier function and, as a consequence, exposure of the mucosa to hydrochloride acid and volatile fatty acids. The production of saliva, including bicarbonate, acts as a buffering capacity for the acidic environment [35]. The saliva production, including the roughage, is found to minimize the occurrence of ESGD by increasing the buffer and providing a protective layer in the stomach [35,36]. In our report, horses that manifested gastrointestinal symptomatology but did not have EGUS also presented higher levels of bicarbonate in saliva than horses with EGUS, pointing out the relation between low values of bicarbonate in saliva and the presence of EGUS.

This study has various limitations. This is a pilot study, and the results should be confirmed in further trials with a large number of horses. In addition, it would be interesting to elucidate the reason for the increased ammonia in saliva with EGUS; for this purpose, to test if the increase in ammonia could be due to an increase in the activity of the enzyme glutaminase, the activity of this enzyme in horses with EGUS should be measured. Furthermore, it would be of interest to measure ammonia in the gastrointestinal juice of horses with EGUS and evaluate if their levels are correlated to those of saliva to clarify the possible mechanism of the increase in ammonia in this sample. In addition, it would be recommended to evaluate the possible use of ammonia and bicarbonate as biomarkers of treatment monitoring, as the presence of ammonia was found to be associated with a delay in the healing of peptic ulcers in rats [11].

## 5. Conclusions

Ammonia and bicarbonate can be measured in the saliva of horses by spectrophotometric assays in a precise and accurate way. In addition, ammonia showed significantly higher values in the saliva of horses with EGUS compared to healthy horses, while bicarbonate was found to be reduced in EGUS horses. Further studies should be undertaken to elucidate the reasons for both analytes’ dynamics and evaluate their possible applications as biomarkers in this disease.

## Data Availability

The data presented in this study are available on request from the corresponding author. The data are not publicly available due to privacy.

## Supplementary Materials

The following supporting information can be downloaded at: https://www.mdpi.com/article/10.3390/metabo14030147/s1, Table S1: Reason for gastroscopy of the 20 animals suspected of Equine Gastric Ulcer Syndrome with noncompatible findings at gastroscopy examination.
