# Supplementary material for: Automated Spectrophotometric Assays for the Measurement of Ammonia and Bicarbonate in Saliva of Horses: Analytical Validation and Changes in Equine Gastric Ulcer Syndrome (EGUS)
Source: Metabolites. 2024 Feb 28;14(3):147. doi: 10.3390/metabo14030147 (PMC10972414; doi:10.3390/metabo14030147)
Supplement: Supplementary file 1 [file metabolites-14-00147-s001.zip › metabolites-2882655-supplementary.pdf]

metabolites

MDPI

Table S1. Reason for gastroscopy of the 20 animals suspected of Equine Gastric Ulcer Syndrome with noncompatible findings at gastroscopy examination.

Horse	Reason for gastroscopy
1	repeated colic
2	behavioural changes
3	repeated colic
4	riding issues
5	colic
6	colic
7	Abnorm behaviour, rearing and bucking
8	colic
9	owner suspects EGUS
10	recurrent colic
11	colic
12	riding issues
13	unspecific colic
14	abnormal behaviour
15	Colic and sand impaction
16	repeated colic
17	tired
18	behavioural changes
19	riding issues
20	riding issues
